# Selection, characterization and in vivo evaluation of novel CD44v6-targeting antibodies for targeted molecular radiotherapy

**DOI:** 10.1038/s41598-023-47891-2

**Published:** 2023-11-24

**Authors:** A. C. L. Mortensen, H. Berglund, L. Segerström, M. Walle, C. Hofström, H. Persson, P.-Å. Nygren, J. Nilvebrant, F. Y. Frejd, M. Nestor

**Affiliations:** 1 Department of Molecular Medicine and Surgery, Karolinska Institutet, Stockholm, Sweden; 2 Department of Immunology, Genetics and Pathology, Science for Life Laboratory (SciLifeLab), Uppsala University, Uppsala, Sweden; 3 Drug Discovery and Development Platform, Science for Life Laboratory (SciLifeLab), Stockholm, Sweden; 4 Department of Immunotechnology, Lund University, Lund, Sweden; 5 Department of Protein Science, KTH Royal Institute of Technology, Stockholm, Sweden

**Keywords:** Cancer therapy, Drug development, Radiotherapy, Targeted therapies

## Abstract

Molecular radiotherapy combines the advantages of systemic administration of highly specific antibodies or peptides and the localized potency of ionizing radiation. A potential target for molecular radiotherapy is the cell surface antigen CD44v6, which is overexpressed in numerous cancers, with limited expression in normal tissues. The aim of the present study was to generate and characterize a panel of human anti-CD44v6 antibodies and identify a suitable candidate for future use in molecular radiotherapy of CD44v6-expressing cancers. Binders were first isolated from large synthetic phage display libraries containing human scFv and Fab antibody fragments. The antibodies were extensively analyzed through in vitro investigations of binding kinetics, affinity, off-target binding, and cell binding. Lead candidates were further subjected to in vivo biodistribution studies in mice bearing anaplastic thyroid cancer xenografts that express high levels of CD44v6. Additionally, antigen-dependent tumor uptake of the lead candidate was verified in additional xenograft models with varying levels of target expression. Interestingly, although only small differences were observed among the top antibody candidates in vitro, significant differences in tumor uptake and retention were uncovered in in vivo experiments. A high-affinity anti-CD44v6 lead drug candidate was identified, mAb UU-40, which exhibited favorable target binding properties and in vivo distribution. In conclusion, a panel of human anti-CD44v6 antibodies was successfully generated and characterized in this study. Through comprehensive evaluation, mAb UU-40 was identified as a promising lead candidate for future molecular radiotherapy of CD44v6-expressing cancers due to its high affinity, excellent target binding properties, and desirable in vivo distribution characteristics.

## Introduction

The development of antibody-based therapeutics has been remarkably successful in generating efficacious anti-cancer therapies and new formats and targets are continuously explored^1,2^. Over the past decades, the field of antibody-based therapeutics has been evolving rapidly beyond standard monoclonal antibodies with the introduction of e.g., immune cell engagers, checkpoint inhibitors, dual specificity bispecific antibodies, antibody–drug conjugates (ADCs) and antibody-based radiopharmaceuticals i.e., molecular radiotherapy^3,4^.

Molecular radiotherapy combines the advantages of systemic administration with the potency of localized ionizing radiation. By targeting a cancer-associated antigen or structure, a carrier conjugated with a therapeutic radionuclide can mediate the accumulation of radioactivity on cancer cells resulting in a targeted localized radiotherapy. To date, the most widely used molecular radiotherapies are based on the use of small molecules or peptides as target binding units^5,6^. In recent years, several antibody-based molecular radiotherapy candidates have been developed (e.g., ^177^Lu-TLX591 targeting prostate specific membrane antigen (PSMA), ^177^Lu-TLX250 targeting carbonic anhydrase IX and ^131^I-3F8 targeting disialoganglioside) and a selection are currently in late-stage clinical trials^7–10^.

The successful implementation of targeted molecular radiotherapy relies on the specific association or overexpression of the target on cancer cells compared to normal tissues. CD44 is a type I transmembrane glycoprotein that binds hyaluronic acid. Alternative splicing of exons encoding a part of the extracellular domain can generate a range of highly glycosylated CD44 variants (CD44v)^11–16^. CD44v6, the isoforms that contain the region encoded by variable exon 6, has been proposed as a target for molecular radiotherapy^15^. In contrast to standard CD44, which is expressed throughout most tissues, the splice variants are much more tissue specific, and CD44v6 expression is limited to subsets of epithelia and during specific developmental stages^14,15^. CD44v6 is overexpressed in several cancers, including head and neck squamous cell carcinoma (HNSCC), ovarian, colorectal, and thyroid cancer. High CD44v6 expression often signifies a poor prognosis, aggressive disease, and increased invasive and metastatic capability^17–21^. Given its limited expression in healthy tissues and its prominent overexpression in various cancer types, CD44v6 has been considered a promising target for molecular radiotherapy, and previous clinical experience suggest usefulness both from a safety and an efficacy perspective^15,16,22^. However, to further improve the therapeutic efficacy, new fully human, higher affinity binders conjugated to a different radionuclide are warranted.

The aim of this study was to generate and characterize a panel of human anti-CD44v6 antibodies. This involved conducting in vitro analyses and in vivo biodistribution studies using xenograft models expressing high levels of CD44v6. The objective was to identify a promising candidate for future development of a molecular radiotherapy drug specifically targeting CD44v6-expressing cancers.

## Results

### Antibody generation and binding screens

To identify novel antibodies to CD44v6, in-house constructed human Fab and scFv phage libraries were used in in vitro selections. These selections involved both negative selection to CD44iso6 as well as positive selections to CD44iso4, to enrich for binders specifically targeting the 43 amino acid v6 region of CD44v6^23,24^. Following phage display selections, a total of 1165 clones from panning rounds 3 and 4 were individually expressed and characterized in a panel of binding assays. The stepwise screening procedure of ELISA, sequencing, HTRF and affinity screening by SPR funneled down the number of candidate clones to 14 lead candidates (11 scFv and 3 Fab). A majority of the 14 clones demonstrated binding to CD44v6, with apparent affinities towards a CD44iso4-Fc fusion construct (Table 3, see “Materials and methods” section) in the low nanomolar range (data not shown). Also, binding to the peptide mimicking the v6 region was seen both by ELISA and HTRF (data not shown). In contrast, with the exception of one clone (UU-84), no binding was detected for CD44iso6, which does not contain the v6-binding region, illustrating that the subtractive panning on the related v6-negative CD44 isoform was successful. Additionally, the top six candidates (UU-8, UU-10, UU-40, UU-44, UU-46 and UU-86) were evaluated as either scFv or Fab clones for binding to both cynomolgus and mouse CD44v6. As expected, cross-reactivity binding was demonstrated toward cynomolgus CD44v6 but not mouse CD44v6 (Supplemental Fig. 3).

### Conversion to hIgG4 and subsequent binding and biophysical characterization

**Figure 1:**
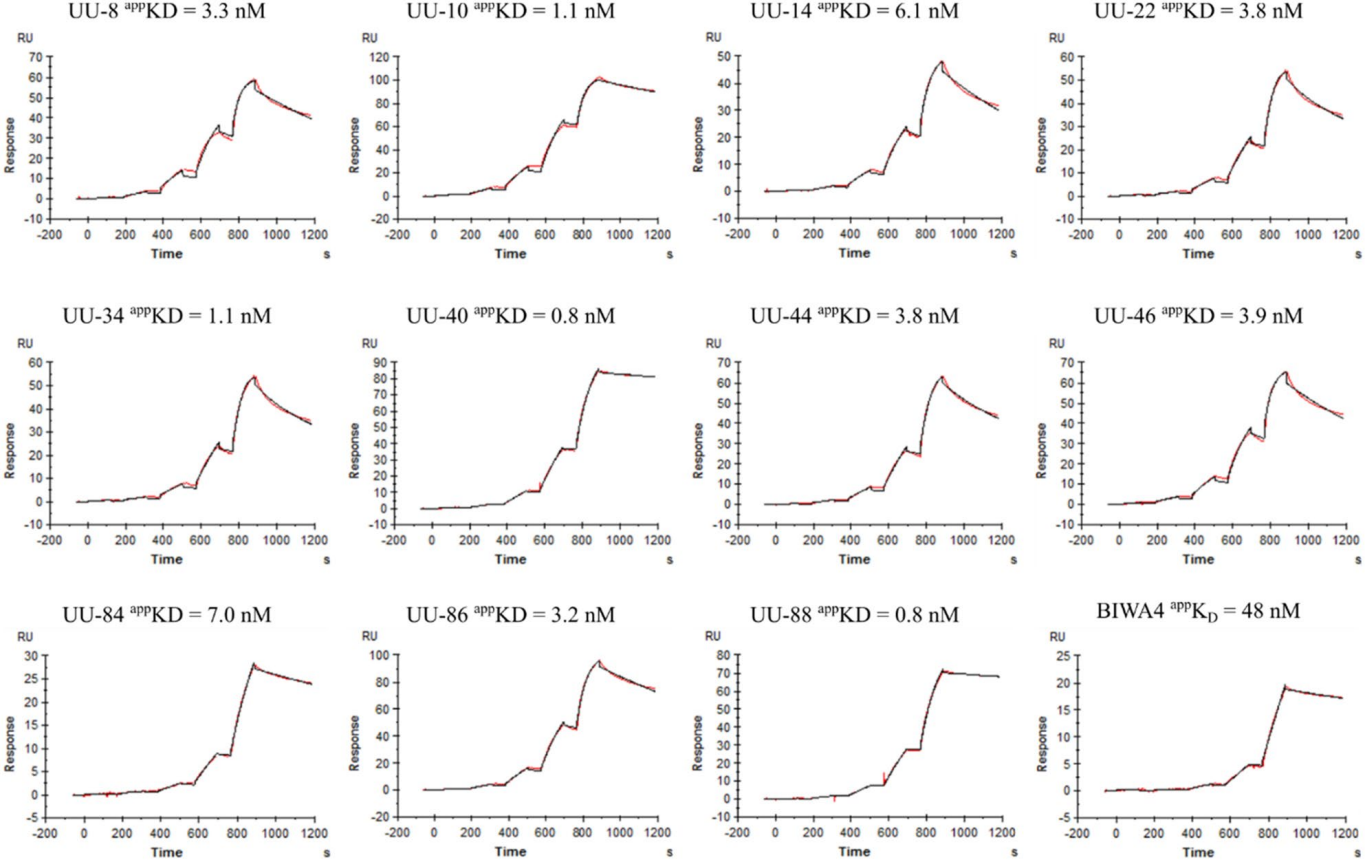
Kinetic measurements: single cycle kinetics sensorgrams of a selection of the generated anti-CD44v6 IgG4 antibodies using SPR. Each antibody was captured by an immobilized anti-Fab antibody following injection of CD44iso4, in a fivefold serial dilution between 80 and 0.3 nM. Red lines represent the measured curves, while the black lines represent 1:1 kinetic binding model curve fits. ^app^K_D_ rather than K_D_ is given for all these measurements to highlight a potential avidity contribution as a result of the dimeric form of the target protein used (Fc-fused).

Following conversion to human IgG4, all UU-clones, with the exception of UU-126, as well as the positive control BIWA4, displayed retained binding towards CD44v6 (Fig. 1). Apparent affinities of the IgG4 UU-antibodies to CD44iso4 were in the low-to-sub nanomolar range, several of which bound with greater affinities than BIWA4.

**Figure 2:**
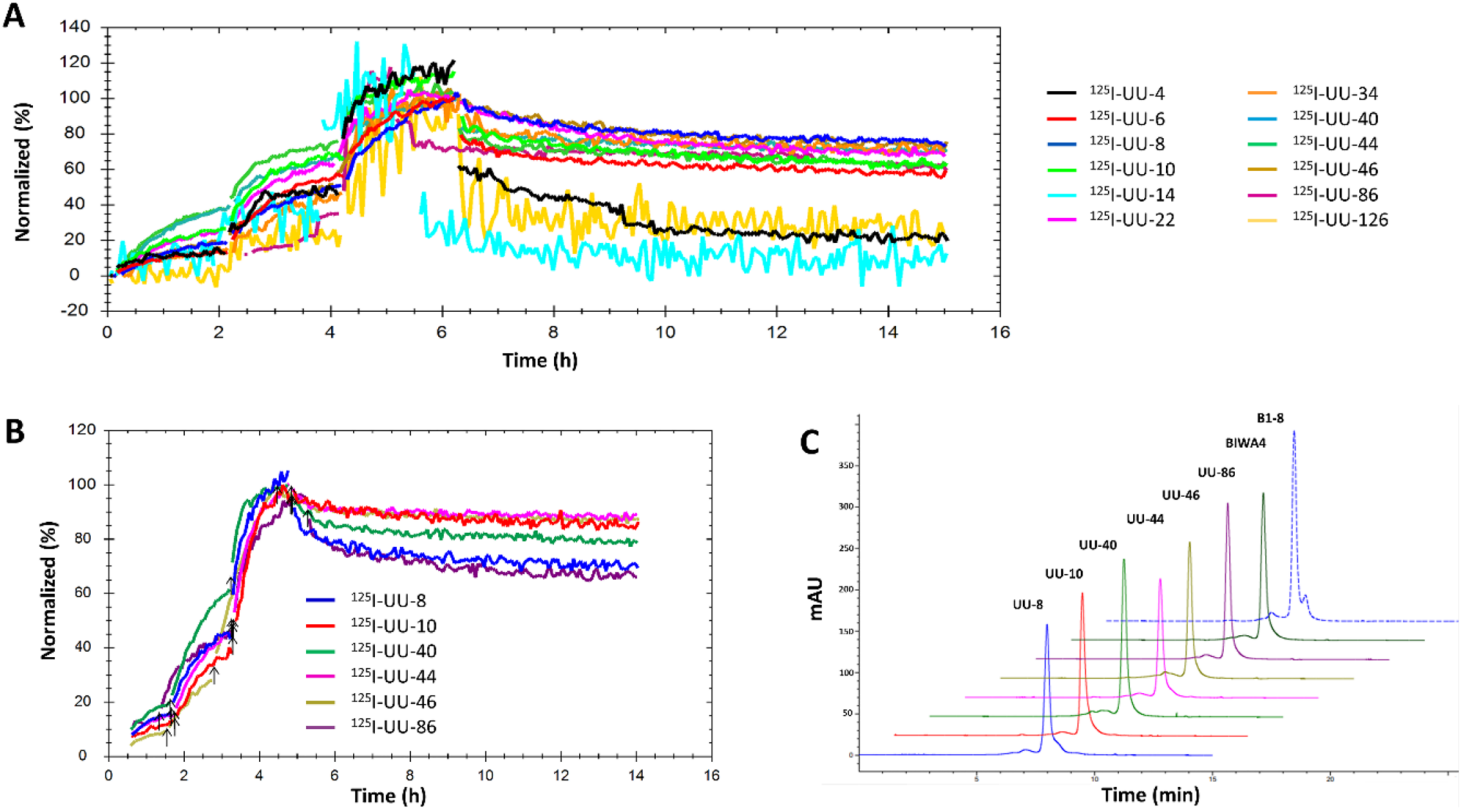
(**A**) LigandTracer analyses of ^125^I-UU antibody candidates on A431 cells (high CD44v6-expressing squamous cell carcinoma line). (**B**) LigandTracer analysis of hIgG4 antibodies (1, 3 and 10 nM) labeled with ^125^I, on ACT-1 cells (high CD44v6-expressing anaplastic thyroid cancer cell line). (**C**) Analytical SEC of top six UU-antibody candidates, positive control BIWA4 and negative control antibody, B1–8.

In order to analyze target binding on live cells a selection of IgG4 candidates were labeled with ^125^I and assessed via LigandTracer analyses. The LigandTracer evaluation on A431 cells of ^125^I-labeled UU-antibody candidates identified UU-8, UU-10, UU-22, UU-34, UU-40, UU-44 and UU-46 as the most promising candidates with high responses and slow off-rate kinetics, whereas poor cell binding was observed for UU-4, UU-14 and UU-126 (Fig. 2A). Interestingly, this cell binding assay showed that there was not a strict correlation between SPR affinity and effective cell binding.

To assess potential off-target binding, the IgG-converted clones were challenged towards a panel of diverse antigens. The off-target binding ELISA results favored UU-14, UU-40 and UU-86, which all generated low signals to the panel of antigens, whereas partial binding was observed and thus an increased risk of unfavorable off-target binding indicated for UU-4, UU-6, UU-8, UU-10, UU-22 and UU-36 (Table 1, Supplemental Figs. 1 and 2).

**Table 1:** Summary of physio-chemical properties of top six UU-IgG4 antibodies.

Name	^app^K_D_ (nM) (SPR)	^app^K_D_ (nM) (cells)	Monomericity (%)	Titer (mg/L culture)	Off-target binding
UU-8	3.3	0.21	> 90	45	Medium
UU-10	1.1	0.12	> 90	16	Low
UU-40	0.8	0.098	> 90	63	Low
UU-44	3.8	0.065	> 90	33	Medium
UU-46	3.9	0.147	> 90	61	Low
UU-86	3.2	0.21	> 90	51	Low

Calculated ^app^K_D_ SPR values were calculated from data as presented and described in Fig. 1. K_D_ on cells (ACT-1 cells) were based on measurements using a 1:1 binding model in the TraceDrawer software.

Based on a thorough evaluation of the results also from the orthogonal characterization assays, six promising candidates (UU-8, UU-10, UU-40, UU-44, UU-46 and UU-86) were selected for in vivo analyses. LigandTracer analyses of ^125^I-labeled top UU-antibody candidates on ACT-1 cells reveled only minor differences and excellent cellular retention for all candidates (Fig. 2B, Table 1). SEC chromatograms of the hIgG4 antibodies demonstrated that they were all primarily monomeric with low degrees of high molecular weight aggregates (Fig. 2C), which indicates that cell binding results were not influenced by antibody self-association.

### In vivo evaluation of the top six anti-CD44v6 IgG4-antibody candidates

**Figure 3:**
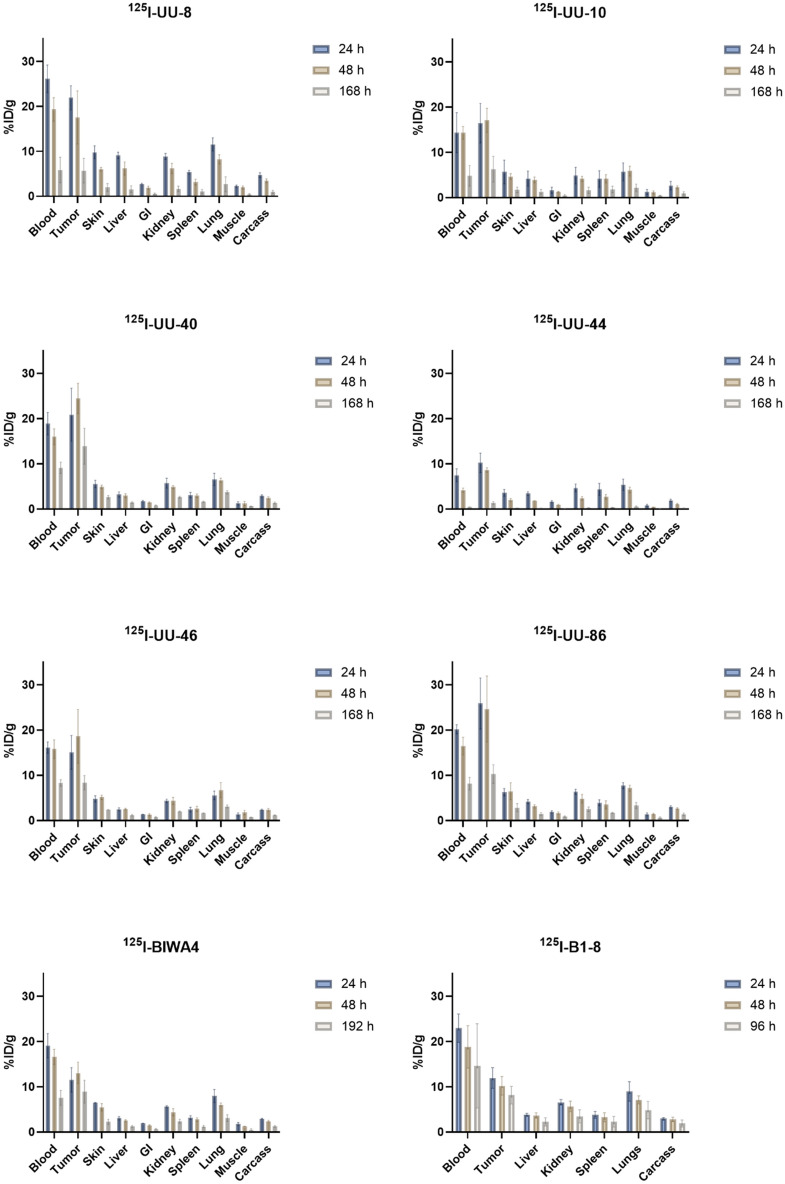
Biodistribution in mice carrying ACT-1 xenografts of top six ^125^I-UU IgG4 antibodies at 24 h, 48 h and 168 h p.i., ^125^I-BIWA4 (positive control) at 24 h, 48 h and 192 h p.i. and ^125^I-B1-8 (negative isotope control) at 24 h, 48 h and 96 h p.i. Error bars represent SD, n ≥ 3 per time point and group.

To determine in vivo behavior and potential differences between the top six candidates, biodistribution studies in xenograft-bearing mice were performed using ^125^I-labeled antibodies. Peak uptake for all antibodies were at either 24 h or 48 h p.i., decreasing at 168 h p.i.^125^I-UU-44 demonstrated the lowest peak tumor uptake, while ^125^I-UU-40 and ^125^I-UU-86 provided the highest, with peak tumor uptake of 25%ID/g (Fig. 3).

**Figure 4:**
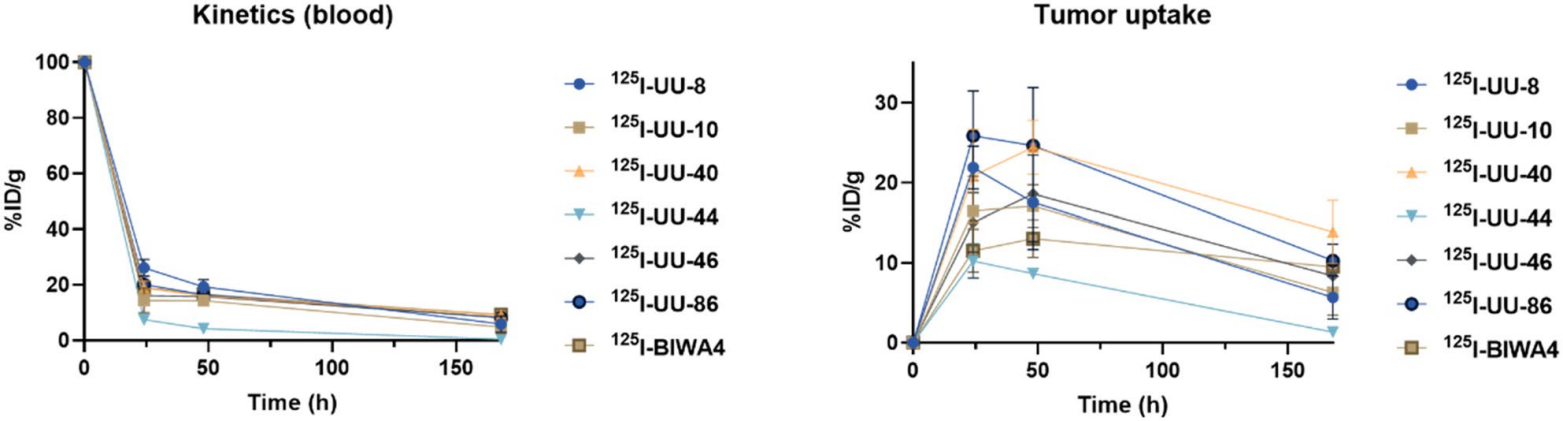
Left: Blood kinetics of ^125^I-UU antibodies and ^125^I-BIWA4 as %ID/g, and Right: tumor uptake of ^125^I-UU antibodies and ^125^I-BIWA4 presented as Area under curve (AUC of %ID/g), assuming that at time = 0 h, 0% of the %ID is in the tumor. Error bars represent SD, n ≥ 3 per time point and group.

The low uptake of ^125^I-UU-44 was attributed to a faster clearance of the antibody from circulation, where less than 1%ID/g remained 168 h p.i., compared to 4–9%ID/g for the remaining candidates (Fig. 4, left). Lower tumor retention was observed for ^125^I-UU-8 and ^125^I-UU-10, where approximately one third of the peak activity remained in the tumors at 168 h p.i. The highest tumor retention was observed for ^125^I-UU-40, with approximately 60% of the peak uptake retained within the tumors at the final time point. The positive control antibody, ^125^I-BIWA4, displayed high tumor retention, albeit with a noticeably lower peak uptake compared to ^125^I-UU-40, which emerged as the most favorable candidate (Fig. 4, right, Table 2). The radiolabeling yield and method (i.e., Pierce Iodination Beads, ≥ 95%, or Pierce Iodination Tubes, ≥ 99%) did not influence the biodistribution results.

**Table 2:** Comparison of %ID/g as AUC, assuming at time = 0, 0% of the injected activity is in the tumor, of ^125^I-UU-40 and ^125^I-BIWA4 in ACT-1 xenografts, including 95% Confidence intervals (CI).

Antibody	^125^I-UU-40	^125^I-BIWA4
AUC (SD)	3093 (328.5)	1784 (152.1)
95% CI	2449–3737	1486–2083

n ≥ 3 per time point and group.

### UU-40 validation in additional xenograft models

**Figure 5:**
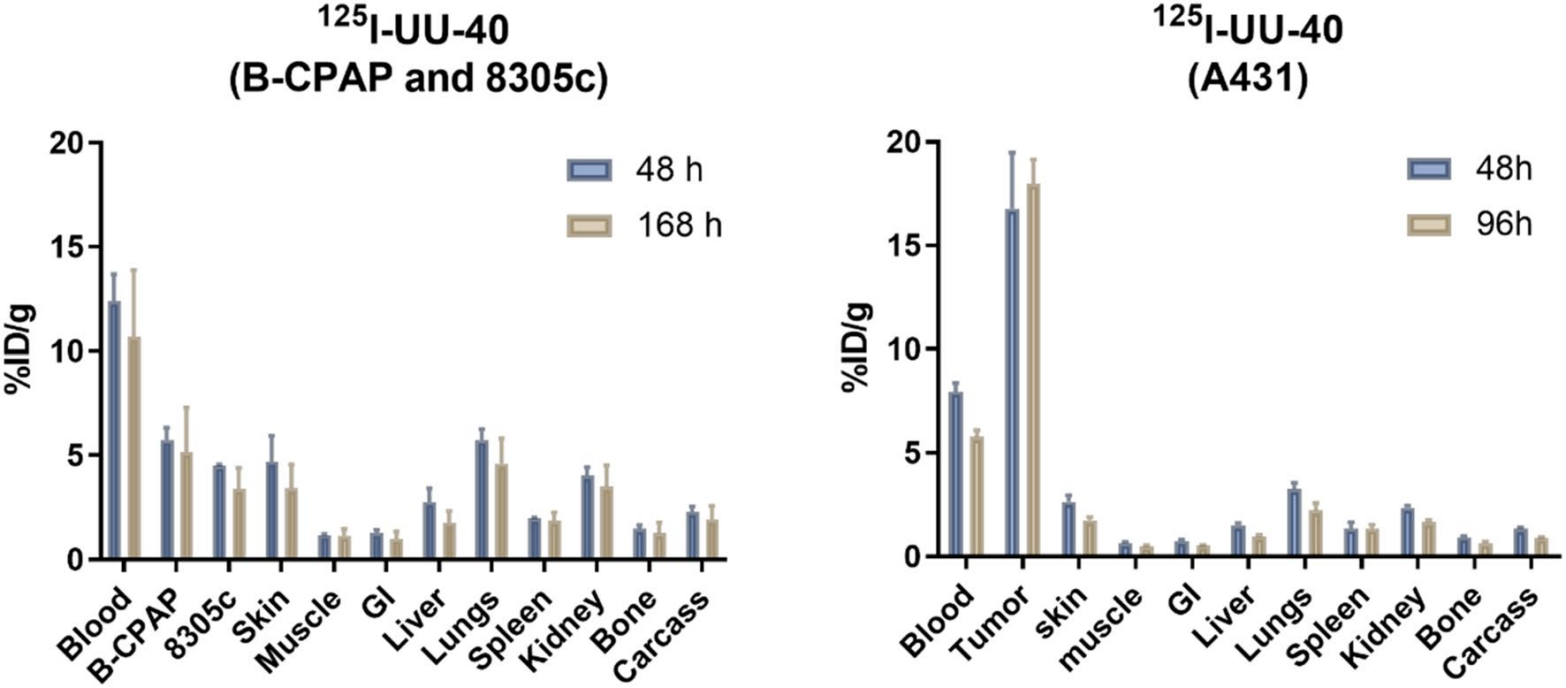
Left: Biodistribution of B-CPAP and 8305c of ^125^I-UU-40 at 48 h and 168 h p.i. (low CD44v6-expressing xenograft models) and Right: biodistribution of ^125^I-UU-40 in A431 xenografts (high CD44v6-expressing xenograft model) at 48 h and 96 h p.i. Error bars represent SD, n ≥ 3 per time point and group.

Evaluation of in vivo specificity of ^125^I-UU-40 was validated in additional xenograft models with varying expression levels of CD44v6 (Fig. 4): the high CD44v6-expressing A431 xenograft model and the low CD44v6-expressing xenograft models, B-CPAP and 8305c. B-CPAP and 8305c have an estimated 10% of the antigen expression levels of A431 and tumor uptake was reflected in this, with peak uptakes of 5.7 ± 0.6%ID/g and 4.5 ± 0.06%ID/g for B-CPAP and 8305c, respectively (Fig. 5, left). A431 xenografts amassed similar uptake levels as observed earlier for ACT-1 xenografts (Fig. 5, right), albeit a slightly lower peak uptake (18 ± 1.2%ID/g).

Taken together, the newly developed anti-CD44v6 antibody UU-40 shows excellent specificity towards CD44v6 both in vitro and in vivo and favorable drug-like properties, illustrating the premise of its future use within the field of molecular radiotherapy.

## Discussion

The primary objective of this study was to identify and characterize novel anti-CD44v6 antibodies for potential use in molecular radiotherapy. Synthetic phage display libraries of human scFv and Fab antibody fragments developed at SciLifeLab and their associated Drug Discovery and Development platform (DDD) were utilized for this purpose. Via sequential in vitro and in vivo characterization steps, including biodistribution studies in xenograft-bearing mice, a lead candidate with promising properties for molecular radiotherapy of CD44v6-expressing cancers was identified. The study illustrates the importance of performing several orthogonal assays to assess e.g., affinity, off-target binding, tolerance to labeling, cellular retention and biodistribution for pinpointing antibodies suitable for further development in a given therapeutic context.

Utilizing the CD44v6 cell surface antigen for molecular radiotherapy carries the advantage of potentially targeting numerous cancers, particularly those derived from epithelial tissues. Lutathera® and Pluvicto® both target highly cancer-specific targets (somatostatin receptor (SSTR) and PSMA, respectively) but target expression is limited to specific forms of cancer, primarily those of neuroendocrine origin that tend to express SSTR, and prostate cancers expressing PSMA. The overexpression of CD44v6 in numerous solid cancers, along with its limited distribution in normal tissues, highlights the potential of CD44v6 as a target for molecular radiotherapy. However, despite previous exploration, the full realization of CD44v6 as a molecular target is yet to be achieved. In 2003, Börjesson et al. evaluated the humanized monoclonal antibody BIWA4 (bivatuzumab) labeled with ^186^Re in a phase I clinical study in HNSCC patients, yielding promising results^16^. The study emphasized the benefits of highly specific monoclonal antibodies, the promise of molecular radiotherapy in solid cancers, and validated the safety and potential of CD44v6 as a target for molecular radiotherapy. Despite these results, the strategy for ^186^Re-BIWA4 was abandoned in favor of an ADC (bivatuzumab mertansine). When the ADC was evaluated in HNSCC patients, it led to adverse events due to skin toxicity, a direct result of bivatuzumab mertansine binding to CD44v6 present on skin keratinocytes^25^. Contrary to ADCs and other cytotoxic compounds, the therapeutic mechanism of action in molecular radiotherapy is the deposition of ionizing radiation along a path chartered by the electron. Therefore, the thin lining of cells expressing CD44v6 in normal tissues may be less susceptible to severe toxicities from molecular radiotherapy than compared with the cytotoxic payload of an ADC, as demonstrated in the ^186^Re-BIWA4 clinical trials where side effects were mild. The majority of the ionizing radiation is deposited outside of the CD44v6-expressing epithelium and, in the case of keratinocytes of the skin, partially outside of the body. Thus, the collective results of the ^186^Re-BIWA4 and bivatuzumab mertansine phase 1 studies support the therapeutic targeting of CD44v6 via molecular radiotherapy as opposed to an ADC approach.

Here, we report the successful selection and thorough clonal characterization of recombinant human monoclonal antibodies that bind specifically to CD44v6. The antibodies were characterized in the hIgG4 format to reduce potential in vivo effector functions like antibody-dependent cellular cytotoxicity (ADCC) and complement-dependent cytotoxicity (CDC) in order to focus on their intended use as radioactive payload carriers.

Generally, several antibody candidates performed similarly across all in vitro evaluations, with low-to-sub nanomolar affinities confirmed both via SPR and on living CD44v6-expressing cancer cells (Figs. 1, 2). Several candidates displayed highly selective, specific and stable target binding and also excellent drug-like physiochemical properties. Binding to CD44v6 on live cells established that while k_on_-rates were similar, several UU-antibody candidates demonstrated poor cellular retention ultimately favoring UU-8, UU-10, UU-22, UU-34, UU-40, UU-44 and UU-46 (Fig. 2A). While no clear lead drug candidate was identified based on the in vitro characterization alone, UU-40 demonstrated superior affinity and low off-target binding (Table 1).

Six UU-antibody candidates were subsequently chosen for in vivo evaluation in tumor bearing mice (^125^I-UU-8, UU-10, UU-40, UU-44, UU-46 and UU-86). The UU-antibodies do not have cross-reactivity with murine CD44v6 (Supplemental Fig. 3) and thus, the biodistribution data does not reflect the distribution into normal tissues expressing CD44v6. However, analyses of the in vivo behavior quickly allowed for the exclusion of three of the candidates due to either poor tumor retention (UU-8, UU-10) or rapid elimination (UU-44) (Fig. 3). The rapid elimination of UU-44 could have been the result of antibody aggregation. However, due to the passive diffusion of ^125^I from the cells following catabolism of UU-44, any potential in vivo aggregation went undetected. Interestingly, both UU-8 and UU-10 were among the candidates with the best cellular retention in in vitro assays, emphasizing the importance of in vivo testing of biological molecules. Of the three remaining candidates (UU-40, UU-46, UU-86), UU-40 demonstrated the most favorable distribution, with high peak tumor uptake and superior retention (Figs. 3, 4). The total tumor uptake was significantly greater for ^125^I-UU-40 than for the positive reference antibody, ^125^I-BIWA4 (Fig. 4, Table 2). Ability to distinguish between high and low CD44v6-expression levels was confirmed through antigen-dependent tumor uptake in tumor bearing mice with varying CD44v6-expression levels (Fig. 5). The collective data suggest that the UU-40 antibody has desirable drug-like characteristics and could have the potential for future use in molecular radiotherapy.

## Conclusion

In this study, we successfully identified high-affinity human anti-CD44v6 monoclonal antibodies using phage display technology. Among the selected antibodies, UU-40 hIgG4 emerged as a promising lead drug candidate, demonstrating favorable in vivo distribution properties in tumor cell xenograft experiments. Further investigations focusing on tumor retention and appropriate radiolabel selection are warranted to validate the potential utility of UU-40 hIgG4 as an antibody-based radiopharmaceutical for targeted therapy of CD44v6-positive cancers. These findings provide a foundation for future development and optimization of UU-40 hIgG4 as a therapeutic agent in the field of targeted molecular radiotherapy.

## Materials and methods

### Phage display selections

Phage display selections were performed with four rounds of enrichment employing in-house constructed human synthetic scFv and Fab phagemid libraries, denoted SciLifeLib, similar in design and construction as previously reported^26^. The v6-positive CD44 isoform 4 (CD44iso4) was used as target with negative selection on CD44 isoform 6 (CD44iso6), which is identical except for a lack of the targeted v6 region CD44 isoforms were produced in HEK293 cells as C-terminal IgG1 Fc fusions by Promab Biotechnologies (Table 3)^12^. The two CD44 isoforms were chemically biotinylated by standard amine coupling. CD44v6 was immobilized onto paramagnetic streptavidin coated beads and parts of the selection process, including the steps of antigen-phage incubation to elution, were automated and performed with a KingFisher Flex (ThermoFisher Scientific). Prior to the phage-antigen incubation step in the three first rounds, the phage stocks were subjected to a negative selection step involving incubation with streptavidin-beads containing immobilized CD44iso6, to deplete binders that did not target the v6-region. The selection pressure was increased in successive rounds by gradually decreasing the antigen concentration and by increasing the intensity and number of washes. Elution, amplification and precipitation of phages was performed essentially as described^27^. To allow for clonal screening of soluble scFv or Fab proteins, phagemid DNA from the third and fourth round of panning was isolated. To allow for screening of soluble scFv or Fab proteins, phagemid DNA from the third and fourth round of panning was isolated, subcloned and individual colonies expressed as previously reported^27^.

**Table 3:** Proteins and peptides used in phage display selections and subsequent binding assays.

Protein	Short name	Uni-prot	Supplier	Format	M_W_ (kDa)	Description
CD44 (isoform 4)	CD44iso4	P16070	Promab Biotech-nologies	Fc-fused, dimeric presentation of CD44iso4	94.4	Naturally occurring v6-positive CD44 isoform^12^
CD44 (isoform 6)	CD44iso6	P16070	Promab Biotech-nologies	Fc-fused, dimeric presentation of CD44iso6	89.5	Naturally occurring CD44 isoform lacking v6-region^12^
Human v6 peptide	v6 peptide		Bachem AG	N-terminally biotinylated	5.0	N-terminally biotinylated, 43 amino acid peptide with sequence QATPSSTTEETATQKEQWFGNRWHEGYRQTPREDSHSTTGTAA
Cynomolgus v6 peptide	n/a	AOA2K5VJEO isoform 1	Bachem AG	N-terminally biotinylated		N-terminally biotinylated, 43 amino acid peptide with sequence QAIPSSTTEETSTQKEQWFGNRWHEGYLQTPREDSHSTTGTAA
Mouse v6 peptide	n/a	P15379 isoform 1	Bachem AG	N-terminally biotinylated		N-terminally biotinylated, 43 amino acid peptide with sequence TPNSTAEAAATQQETWFQNGWQGKNPPTPSEDSHVTEGTTA

### Screening for CD44v6 binding

A total 733 supernatants containing scFv and 433 containing Fab were screened for specific binding to the v6-region of CD44v6 using a stepwise screening procedure of ELISA, Sanger sequencing, homogenous time-resolved fluorescence (HTRF) and affinity screening by surface plasmon resonance (SPR) essentially as previously described^27^. Briefly, in the ELISA biotinylated antigens were added to streptavidin-coated wells. The interaction between antibody fragments and immobilized antigen was detected using horseradish peroxidase (HRP) conjugated anti-FLAG (for scFv clones) or anti-IgG Fd antibodies (for Fab clones). Clones considered positive were subjected to Sanger sequencing. The sequence unique clones were subsequently evaluated in a HTRF energy transfer assay where binding to biotinylated CD44iso4, CD44iso6, v6 peptide (Bachem AG) and a non-related protein was further assessed. Detection of binding was enabled either through a terbium-conjugated anti-FLAG antibody (cat. no. 61FG2TLF, Cisbio) for scFv or using an europium-conjugated anti-kappa (cat. no. 61KAPKAA, Cisbio) for Fab functioning as donor molecule and streptavidin-conjugated XL665 (cat. no. 610SAXLF, Cisbio) as acceptor molecule. Finally, an SPR-based affinity screen was performed using a Biacore T200 instrument (Cytiva). For analyses of scFv clones, an anti-FLAG M2 antibody (cat. no. F3165, Sigma-Aldrich), was immobilized as capture ligand onto all four surfaces of a CM5 Series S sensor chip by amine coupling according to manufacturer´s recommendations. Analogously, for analyses of Fab clones an anti-Fab antibody (Cytiva) was used as capture ligand. Fab or scFv were injected and captured onto the chip surfaces, followed by injection of 50 nM CD44iso4 or 50 nM CD44iso6. The surfaces were regenerated with 10 mM glycine–HCl pH 2.5 (for scFv) or pH 2.1 (for Fab). By subtracting the response to a reference surface with an anti-FLAG or anti-Fab antibody immobilized, response curve sensorgrams for all clones were obtained. Data were analyzed using the Biacore T200 Evaluation 3.1 software and kinetic parameters were calculated assuming a 1:1 Langmuir binding model. The top-performing candidates in the kinetic screen were chosen for more detailed kinetic measurements. The setup was similar to the affinity screening, except that several concentrations of the antigen were injected in single-cycle kinetics mode. The antigen concentrations injected were: 0.16, 0.8, 4, 20, 100 nM of CD44iso4 for the scFv binders, whereas the Fabs were analyzed using 1, 3, 9, 27 and 80 nM of CD44iso4.

### Screening for cynomolgus and mouse CD44v6 binding

384-well ELISA plates were coated with 1 µg/mL streptavidin in PBS for 1 h at 37 °C. Following wash and blocking of plates in block buffer (PBS with 0.5% bovine serum albumin and 0.05% Tween20), biotinylated peptides and CD44iso6 and a non-relevant protein were allowed to bind for 30 min. UU-scFv and Fab clones, present in bacterial supernatant diluted 1:5, 1:20 and 1:80 in block buffer were incubated for 90 min. Detection of binding was enabled using HRP conjugated anti-FLAG (for scFv clones) or anti-IgG Fd antibodies (for the Fab clone). Binding signal development was started by adding TMB ELISA substrate (cat. no. T2885, ThermoFisher Scientific) and terminated by the addition of 1 M H_2_SO_4_. Plates were analyzed at 450 nm on a SpectraMax Plus spectrophotometer (Molecular Devices).

### Conversion to IgG, expression, purification and characterization

Plasmid DNA of 14 CD44v6-specific scFv or Fab selected for IgG4 conversion (UU-4, 6, 8, 10, 14, 22, 34, 36, 40, 44, 46, 86, 88 and 126) was purified from bacterial cultures by a standard miniprep procedure. The previously described anti-CD44v6 mAb BIWA4 was included as positive control^28^. UU-41, which bound to CD44 but not to the v6 region, and the anti-NP (4-hydroxy-3-nitrophenyl) antibody, 1B7, were included as negative controls^29^. Sequences of the variable domains of the heavy (VH) and light (VL) chains of BIWA4 were obtained from patent application WO2002094879. The sequences of the variable domains of the anti-NP antibody, 1B7, were obtained from PDB (1A6V). The VH and VL regions were PCR amplified and inserted into in house constructed vector pHAT-hIgG4 using the In-Fusion HD Plus Cloning Kit (cat. no. NC0473876, Clontech). The gene encodes the IgG4 antibody heavy chain S228P substitution (EU numbering), a stabilizing mutation in the IgG4 hinge region preventing half molecule exchange^30,31^. Transfection of plasmid DNA into expiHEK293 cells was performed using an ExpiFectamineTM 293 Transfection Kit (cat. no. A14525, ThermoFisher) in 4 mL cultures in a 24 deep well plat. After 5 days of cultivation at 37 °C, 6% CO_2_, 80% rH and 400 rpm, the media supernatant was mixed with Protein A conjugated paramagnetic beads and purified on a KingFisher Flex instrument (cat. no. 5400610, ThermoFisher). Immediately following elution in 0.1 M glycine, pH 2.7, neutralization was performed by addition of 1 M Tris–HCl, pH 8.8, and buffer exchange to PBS. SDS-PAGE analysis was performed to determine purity and integrity of the purified IgG4s and concentrations were determined using an Implen NP80 UV–Vis Spectrophotometer (Implen). Analytical size-exclusion chromatography (SEC) was performed to determine the monomeric purity of the six lead UU-antibody candidates (UU-8, UU-10, UU-40, UU-44, UU-46 and UU-86) as well as positive (BIWA4) and negative (B1-8) control antibodies. The pooled desalted Protein A fractions were concentrated using Amicon Ultra-4 (10 kDa cutoff, UFC801024, Sigma Aldrich) centrifugal units. The concentrated antibody pools were loaded on a HiLoad 16/600 Superdex 200 pg SEC column (cat. no. 28-9893-35, Cytiva) equilibrated with PBS. The antibodies were diluted to an approximate concentration of 1 mg/mL per sample and loaded with a flowrate of 1 mL/min. Estimation of mg protein concentration was performed using area under curve and extinction coefficient 1.4.

### Kinetic measurements

SPR experiments of the purified antibodies were run using a single cycle kinetics (SCK) approach. Two different sensor chips were prepared, an anti-human Fab chip and a streptavidin chip, to enable capture of hIgG4 clones or capture of biotinylated human v6-peptide, respectively. The anti-Fab chip was prepared as described above. Purified IgG4 antibodies were injected and captured onto the chip surface. A four-fold dilution series of CD44iso4, consisting of five concentrations ranging between 80 and 0.3 nM, were prepared in running buffer (HBS supplemented with 0.05% Tween20 at pH 7.5) and sequentially injected over the chip surfaces. In addition, a CM5 chip pre-coated with streptavidin (cat. no. 29699621, Cytiva) was used to capture biotinylated human v6-peptide at different RU levels, 20 RU and 250 RU. Five-fold dilution series of the different antibodies, ranging between 50 and 0.08 nM were prepared and sequentially injected. Following a dissociation phase, the chip surfaces were regenerated with 10 mM glycine–HCl, pH 2.1. Reference subtraction and analyses were performed as described above.

### Cross-reactivity ELISA (off-target binding)

Potential off-target binding was tested against baculovirus particles (BVPs) and against a panel of antigens; Cardiolipin (cat. no. SRE0029, Sigma-Aldrich), Keyhole Limpet Haemocyanin (KLH) (cat. no. H7017, Sigma-Aldrich), lipopolysaccharide (LPS) (cat. code tlrl-eblps, InvivoGen), single stranded DNA (ssDNA) and double stranded DNA (dsDNA) from calf thymus (cat. no D8899 and D4522, respectively, Sigma-Aldrich) and human insulin (cat. no. I-034, Sigma-Aldrich) using previously described procedures. BVPs were produced in-house by PEG/NaCl precipitation from the supernatant of baculovirus infected SF9 cells. In the ELISA, the BVPs (1:20), Cardiolipin (50 µg/mL), KLH (5 µg/mL), LPS (10 µg/mL), ssDNA (1 µg/mL), dsDNA (1 µg/mL) and insulin (5 µg/mL) were diluted in PBS and added to the wells of an ELISA plate and incubated overnight at 4 °C. The plates were washed and blocked with PBS 0.5% BSA for 1 h at RT. IgG4 antibodies were diluted to 100 nM and 20 nM in PBS containing 0.5% BSA and added to the plates and incubated for 1 h at RT. A secondary goat anti-human IgG kappa-HRP (cat. no. 2040-05, Southern Biotech) was added and incubated for 1 h prior to TMB (ThermoFisher Scientific) development and stopped with 1 M H_2_SO_4_ and read at 450 nm. Off-target binding of UU-antibodies was compared to off-target binding of reference antibodies and “low” was defined as lower or similar to reference antibodies, while “medium” was defined as higher than reference antibodies.

### Cell culture

Cell lines used in this study included the human anaplastic thyroid cancer cell lines ACT-1 and 8305c, papillary thyroid cancer cell line B-CPAP and the human epidermoid carcinoma cell line, A431. The ACT-1 cell line was originally established by Dr. S. Ohata of Tokushima University (Japan) and cultured in Dulbecco’s Modified Eagle Medium (cat. no. L0102, BioWest) and Ham’s F12 (1:1) (cat. no. L0090, BioWest) with 10% Fetal bovine serum (FBS, F2442, Merck Millipore), 2 mM l-glutamine (cat. no. X0551, BioWest) and antibiotics (100 IU penicillin and 100 µg/mL streptomycin, cat. no. L0018, BioWest)^32^. The 8305c and B-CPAP cell lines were obtained from Deutsche SammLung von Mikroorganismen und Zellkulturen (cat. no. ACC 133 and ACC 273, respectively, DSMZ) and cultured in RPMI 1640 (cat. no. L0503, BioWest) with 20% FBS, 2 mM l-glutamine and antibiotics as mentioned above. The A431 cell line was obtained from the American Type Culture Collection (cat. no. CRL-1555, ATCC) and cultured in DMEM with 10% FBS, 2 mM l-glutamine and antibiotics as mentioned above. All cell lines were cultured at 37 °C and 5% CO_2_ for less than three months.

### Radiolabeling

For LigandTracer analyses (see below) of all antibody candidates, radioiodination was performed using chloramine T labeling (CAT) as previously described^33^. Briefly, CAT and Na_2_SO_5_ (NBS) were dissolved in PBS to 4 mg/mL. Approximately 10–20 µg of antibody was labeled with 2 MBq of ^125^I (cat. no. NEZ033H010MC, PerkinElmer) by incubating the reaction solution with 10 µL of CAT (4 mg/mL) for 60 s on ice before ending the reaction with 20 µL NBS (4 mg/mL). The labeling yield using CAT (≤ 85%) was determined by Instant Thin-Layer Chromatography (ITLC) using 70% acetone (v/v) as the mobile phase. For biodistribution studies, radioiodination of B1-8, UU-8 and UU-46 was performed using Pierce Iodination Beads (cat. no. 28665, ThermoFisher Scientific). In short, the beads were washed in PBS prior to the start of the experiment, where typically 5–10 MBq of ^125^I was added to an Eppendorf tube containing 100–200 µL of PBS and Pierce Iodination Beads. Following a 5 min incubation of the ^125^I with the iodination bead, the reaction solution (PBS and ^125^I) was transferred to an Eppendorf tube containing typically 100 µg of antibody and incubated for at least 15 min with gentle shaking. The labeling yield was determined by ITLC using 0.9% NaCl (w/v) as the mobile phase. Due to inferior labeling yields of Pierce Iodination Beads (≤ 95%) compared to Pierce Iodination Tubes (≤ 99%), radioiodinations of BIWA4, UU-10, UU-40, UU-44 and UU-86 were performed using Pierce Iodination Tubes (cat. no. 28601, ThermoFisher Scientific) according to manufacturer’s instructions. Briefly, a Pierce Iodination Tube was washed once with 1 mL of PBS before adding 50–100 µL of PBS to cover the bottom of the tube. On average, 5–10 MBq of ^125^I was added to the tube and incubated for 6 min with gentle swirling every 30 s. Following incubation, the reaction solution was transferred to an Eppendorf tube containing typically 100 µg of antibody and incubated at 37 °C and 350 rpm in a heat shaker for at least 15 min before determining the labeling yield by ITLC. All ITLCs were analyzed using a Bass 1800 II PhosphoImager System (Fuji).

### LigandTracer analyses

LigandTracer procedures have been described previously and were performed according to manufacturer’s instructions^33^. All LigandTracer experiments were performed on LigandTracer Grey (Ridgeview Instruments). Approximately 5–10 × 10^5^ ACT-1 or A431 cells were seeded on tilted petri dishes at least 24 h prior to the start of each assay and incubated at 37 °C with 5% CO_2_. Radioiodinated antibodies were added in two (1 and 3 nM) or three successive concentrations (1, 3 and 10 nM), and the cell-bound radioactivity was measured at room temperature for at least 90 min for each concentration followed by a final dissociation phase of at least 10 h. Analysis was performed using TraceDrawer version 1.7 (Ridgeview Instruments).

### In vivo xenografts and biodistribution studies

Female Balb/c nu/nu mice (N = 121) were housed under standard laboratory conditions and fed and watered ad libitum. All experiments complied with Swedish law and were performed under permit from the Uppsala Committee of Animal Research Ethics (permit no. C33/16) and in compliance with Swedish legislation. All animal studies were reported according to ARRIVE guidelines. For biodistribution studies of ACT-1 and A431, tumor xenografts were formed by subcutaneous inoculation of approximately 1 × 10^7^ ACT-1 or A431 cells into the right posterior flank, suspended in 100 µL serum-free cell culture medium. For biodistribution studies of 8305c and B-CPAP, xenografts were bilaterally inoculated subcutaneously on both flanks in equal amounts of cells (10^7^ cells) and media of suspension (100 µL). Animals were inspected daily for general appearance and formation of tumors, measured by digital caliper using the formula, (L × W × H) × 0.52, to calculate tumor volume. Each animal was injected with 15 µg of ^125^I-antibody in the tail vein and subsequently anesthetized with a mixture of ketamine and xylazine and euthanized by heart puncture prior to excision and collection of selected organs at designated time points. The collected organs, tumors and blood were weighed and measured in a Wizard 2480 automated gamma counter (cat. no. 2480-0010, PerkinElmer) and compared to a standard of the injection solution in order to determine the % injected dose per gram of tissue (%ID/g). On average, the ACT-1 xenografts were 100–200 mm^3^ at the time of antibody injection, evenly distributed among both time points and different antibody groups to ensure fair assessment and cross-referencing of biodistribution data.

### Statistics

Confidence intervals (95%) determined significance between total %ID/g as total uptake (area under curve), with 0% at time = 0 h for tumor uptake of UU-40 and positive control antibody, BIWA4.

### Supplementary Information

Supplementary Figure 1.

Supplementary Figure 2.

Supplementary Figure 3.

## Data Availability

All data is available upon request. Please contact Anja Mortensen, anja.lundgren.mortensen@ki.se.
