# Supplementary figures and images for: Selection, characterization and in vivo evaluation of novel CD44v6-targeting antibodies for targeted molecular radiotherapy
Source: Sci Rep. 2023 Nov 24;13:20648. doi: 10.1038/s41598-023-47891-2 (PMC10673843; doi:10.1038/s41598-023-47891-2)

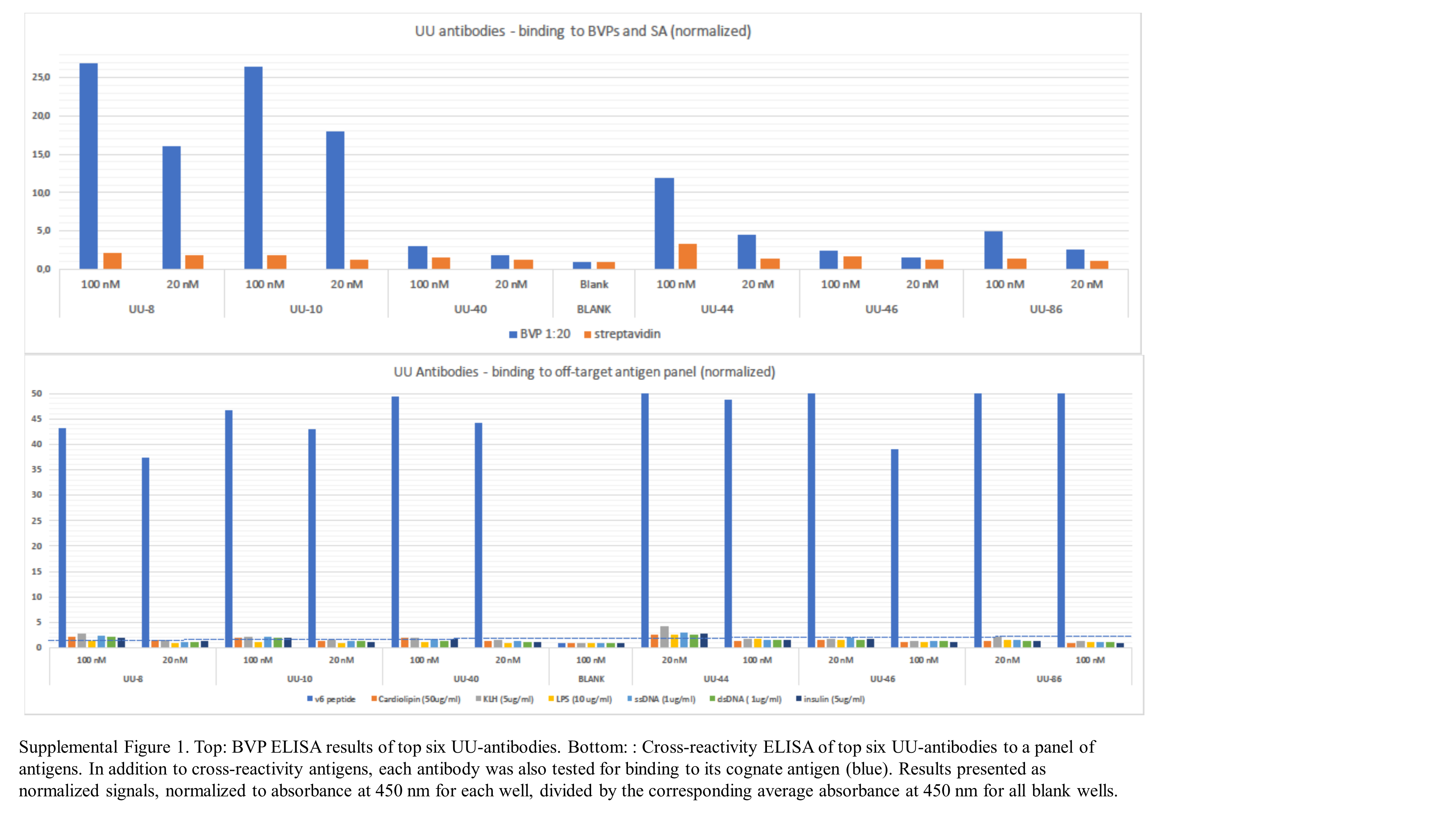

Supplement: Supplementary file 1 — Supplementary Figure 1. [file 41598_2023_47891_MOESM1_ESM.png]

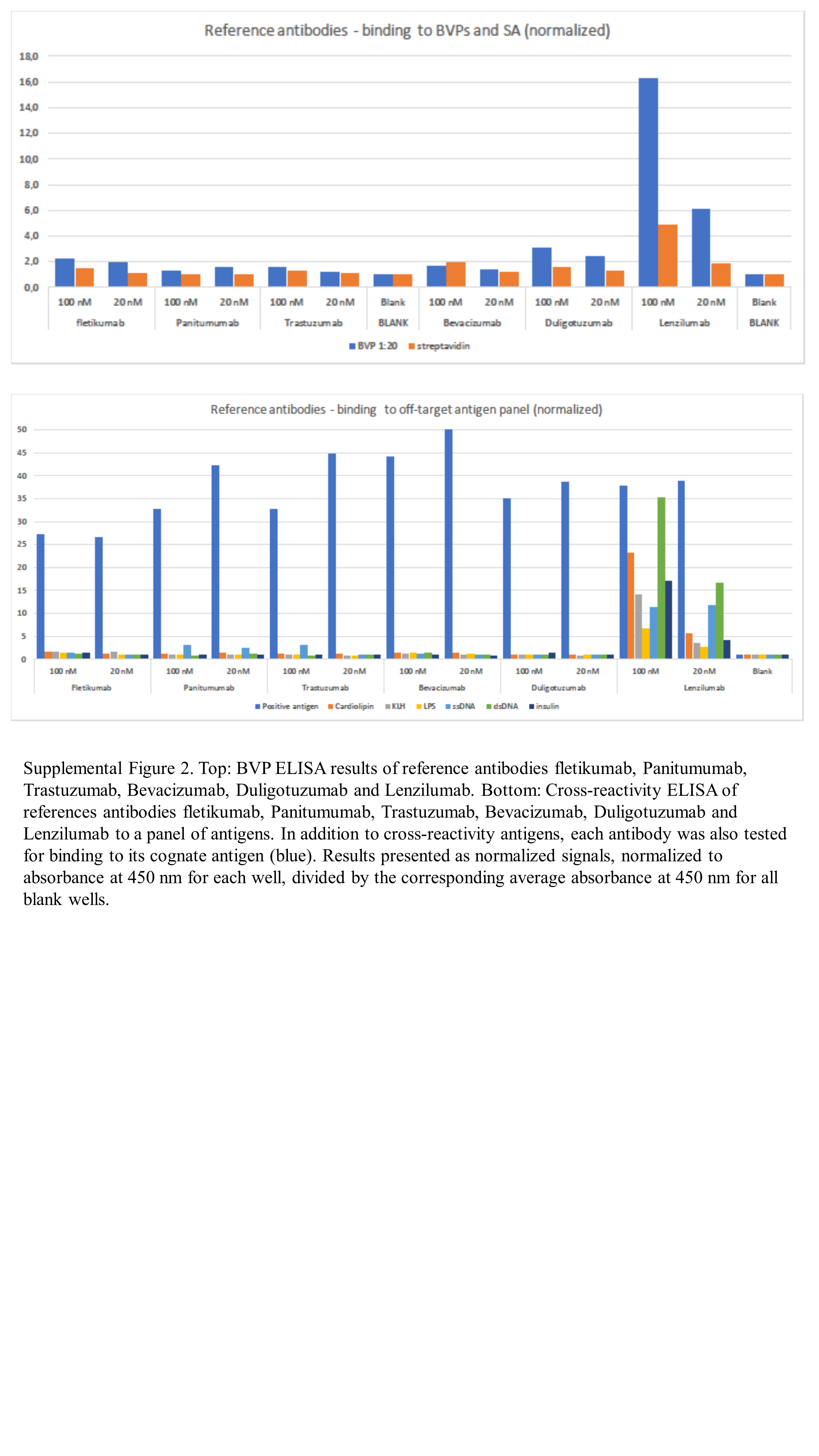

Supplement: Supplementary file 2 — Supplementary Figure 2. [file 41598_2023_47891_MOESM2_ESM.png]
