# Supplementary material for: Selection, characterization and in vivo evaluation of novel CD44v6-targeting antibodies for targeted molecular radiotherapy
Source: Sci Rep. 2023 Nov 24;13:20648. doi: 10.1038/s41598-023-47891-2 (PMC10673843; doi:10.1038/s41598-023-47891-2)
Supplement: Supplementary file 3 — Supplementary Figure 3. [file 41598_2023_47891_MOESM3_ESM.pdf]

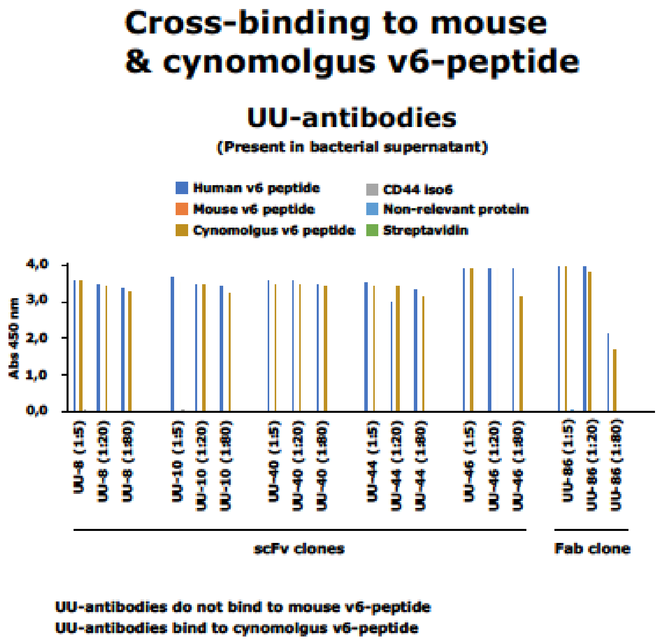

Supplemental Figure 3. Binding signal (absorbance at 450 nm) towards human v6-peptide, mouse v6-peptide, cynomolgus v6-peptide, CD44iso6 (negative control), non-relevant protein and streptavidin of top six UU-candidates as scFv (UU-8, UU-10, UU-40, UU-44 and UU-46) or Fab (UU-86) clones present in the bacterial supernatant and diluted to 1:5, 1:20 or 1:80.
